# A Comprehensive Review on the Utilization of Recycled Waste Fibers in Cement-Based Composites

**DOI:** 10.3390/ma14133643

**Published:** 2021-06-29

**Authors:** Yang Ming, Ping Chen, Ling Li, Guoxing Gan, Gelin Pan

**Affiliations:** 1 College of Civil and Architecture Engineering, Guilin University of Technology, Guilin 541004, China; 2019049@glut.edu.cn; 2 Guangxi Key Laboratory of New Energy and Building Energy Saving, Guilin University of Technology, Guilin 541004, China; 3 Guangxi Engineering and Technology Center for Utilization of Industrial Waste Residue in Building Materials, Guilin 541004, China; ggx18737699886@sina.com (G.G.); pangelin137@sina.com (G.P.); 4 Guangxi Beibu Gulf Engineering Research Center for Green Marine Materials, Guilin 541004, China

**Keywords:** recycled waste fiber, cement-based composites, mechanical properties

## Abstract

Ecological problems such as natural resource depletion and massive quantities of waste for disposal are now guiding progressive civilization towards sustainable construction. The reduction of natural resources and the discarding of debris into open landfills are the two main environmental concerns. As a result, managing these solid wastes is a major challenge worldwide. In comparison to disposal, insufficient landfills, ecological degradation and the economic load on the relevant agencies, recycling and reusing waste materials have a considerable influence. Waste fiber has been studied for use as a cement-based composite (CBC) ingredient. Recycling waste fibers not only makes the cement composite more cost-effective and long-lasting but also helps to reduce pollution. Plastics, carpets and steels are among the various types of waste fibers reviewed in this study for their applications in cement-based materials. The mechanical properties of CBCs with different kinds of recycled-waste fibers were explored, including their compressive, flexural and splitting tensile strength and durability properties. The use of recycled fibers in the construction industry can help to ensure sustainability from environmental, economic and social standpoints. As a result, additional scientific research is needed, as well as guidance for more researchers and experts in the construction sector to examine the unknown sustainability paths. The barriers to the effective implementation of waste fiber recycling techniques in the construction sector were reviewed, and various solutions were proposed to stimulate and ensure their use in CBCs. It was concluded that CBCs containing recycled fibers provide a long-term and cost-effective alternative for dealing with waste materials.

## 1. Introduction

### 1.1. Recycled-Waste Fibers in Cement-Based Composites

Concrete is a composite material composed of various constituents, including cement, sand, coarse aggregates, additives and water [1]. Conventional concrete has shortcomings in terms of its tensile strength, ductility, energy absorption, shrinkage cracking and crack resistance [2,3,4]. In order to address these shortcomings, concrete mixtures have begun to incorporate various types of fibers [2,5]. The incorporation of fibers in concrete is generally intended to improve its mechanical performance for many applications, such as bridge decks, concrete roads, slabs and buildings [6,7,8]. However, recycled-waste fibers are gaining the attention of researchers to enhance the performance of concrete. Fibers are frequently used in the production of lightweight cement-based composite (CBCs), which have a minimal density and a higher thermal insulation capacity, and self-compacting concrete (SCC), which can attain good compaction devoid of mechanical vibration [9]. In civil engineering applications, fiber-reinforced composites (FRCs) are mostly used. Glass fibers are generally preferable for pre-cast members, sewer pipes, wall plaster, curtain wall facings, concrete blocks and thin concrete shell roofs. Steel fibers (SFs) are frequently used in tunnel linings, airport runways, blast-resistant structures, bridge decks, pipes, pavements, floor slabs, roofs and pressure vessels. Synthetic fibers are frequently used in facing panels and piles. Other applications include dam and well construction, tunnel concrete lining, and the stabilization of slopes. Synthetic fibers enhance the performance of concrete, but they are derived from nonrenewable and costly natural resources [10]. Moreover, synthetic fibers are not decomposable and, when dumped, produce waste and have an adverse influence on the environment. On the other hand, natural fibers are more economical and renewable, making them a sustainable source of fibers for FRCs [11,12,13]. By utilizing recycled fibers (RFs), the impact of the concrete industry on the environment, waste disposal, and waste streams in landfills may be reduced. Earlier research has demonstrated that different kinds of fibers recycled from various waste deposits are appropriate for the production of FRCs, and are more economical than synthetic fibers [3]. This increasing use of waste in the production of FRCs bolsters strategies for “closing the loop” in the execution of circular economy practices [14]. From this vantage point, waste material reuse and recycling can be effectively applied to the construction industry. [14].

### 1.2. Mechanical Properties of Fiber-Reinforced Composites

The addition of RFs to concrete may be a step towards making the material more environmentally friendly. Plastic packaging production is a significant contributor to the total municipal waste generated. Plastic waste is non-biodegradable, and is disposed of in landfill sites, where it negatively impacts the environment [15]. Efforts have been made to generate fibers from plastic waste, and to use them in concrete production. The impacts of plastic waste fibers on the performance of CBCs have been studied in a variety of ways. According to some studies, using 1–1.5% waste plastic fiber improves the flexural strength (FS) by about 70%, while others report decreased strength as the waste plastic fiber content increases [16,17,18,19]. Due to concrete’s high consumption and the importance of recycling, the utilization of waste RFs in CBCs has been recommended, and has earned researchers’ interest [20,21,22,23,24]. The application of waste recycled steel fiber (WRSF) to enhance the mechanical properties (MPs) of FRCs has been reported [20,21,22,23,24]. Recently, the influence of WRSF on the bending, tensile and post-cracking behavior of concrete was investigated, and the results revealed that the incorporation of WRSF enhanced the residual strength of concrete [24]. Utilizing waste tire crumb rubber and recovered coarse aggregate in concrete preparation contributes to the construction industry’s long-term sustainability [25]. Furthermore, the research on the mechanical properties and durability of WRSF-reinforced CBCs has revealed that the addition of WRSF improved the impact resistance, shrinkage behavior and crack propagation of concrete compared to that of plain concrete [26,27,28,29]. A recent review concluded that the use of WRSF, which accounts for the majority of the improvements in concrete behavior associated with industrial steel fiber (ISF), is feasible [28]. Several studies have been performed to determine the impact of waste recycled plastic fiber (WRPF) incorporation on the splitting-tensile strength (STS) of concrete composites, and they found improvement in the STS of composites [30,31,32,33]. The incorporation of waste recycled carpet fibers (WRCFs) in cementitious composites resulted in a decrease in their compressive strength (CS) [34,35,36]. Numerous pieces of research have been performed to determine the effect of WRPFs on the FS of cementitious composites. Several researchers reported an overall improvement in FS [32,37,38,39,40,41]. In contrast, a decrease in FS was noticed with the addition of WRPF [42,43]. Generally, WRCF increases the STS properties of the composites to a certain extent [44,45].

### 1.3. Effect of Waste Fiber-Reinforced Composites on the Environment

One of the century’s major challenges is to adopt a sustainable development direction in order to balance current and future generations’ environmental, economic and social needs [46]. From this perspective, the construction sector is experiencing a critical period to satisfy triple bottom line sustainability standards [47,48]. Taking the environmental aspect into consideration, the construction industry consumes half of all raw materials and industrial waste, and accounts for 40% of global energy consumption and 40–50% of greenhouse gas emissions [49,50,51]. Moreover, the industry contributes to environmental concerns such as eco-system deprivation, and water, air and soil contamination [1]. Concrete is an extensively used material in construction, and it plays a critical part in ensuring the global population’s health and safety, being the second most used resource after water [52,53]. Indeed, when appropriately prepared, concrete exhibits remarkable MPs and durability properties, making it an admirably engineered material [54]. Presently, the production of cement is around 4.4 billion tons per year worldwide, but this figure is estimated to be more than 5.5 billion tons by 2050, with the fastest growth in developing countries [55]. Concrete production consumes a large amount of raw resources and contributes significantly to global greenhouse gas emissions. Concrete’s durability performance is critical for allowing the material to reach its intended service life and avoid premature replacement because it is placed in a variety of damage-inducing conditions [56]. As the most widely used building material, concrete needs a global strategy to decrease its effect on the environment and natural resources [57]. The adverse impact of concrete is mainly due to the manufacturing of cement, which is its primary component. Cement accounts for about 5 to 7% of global anthropogenic CO_2_ releases and 3% of global greenhouse gas emissions [1,58]. Additionally, the specific environmental effects, the excessive utilization of natural resources and fossil fuels, and the high level of waste produced must be considered [49]. In order to reach a balance between industrial growth and the planet’s structural constraints, maintaining the competitiveness of this building material, it is required of us to determine solutions for the reduction of the environmental impact of concrete, and to develop new “green” concrete principles [58]. Synthetic plastics are commonly disposed, polluting the environment. To address this issue, plastic waste is recycled as fiber in concrete [59]. In this regard, the concrete industry must move away from the straight route of traditional production and consumption, and towards a rounded methodology which considers the entire construction lifecycle [60]. Given the industry’s critical role in global economic, social, and environmental growth, it can perform a vital role in attaining the United Nations’ Sustainable Development Goals [61]. Many of the 17 sustainable development objectives will somehow be tackled in the growth of sustainable solutions for the sector with the concrete supply chain. The key goals of these techniques are to reduce raw material use by utilizing waste and recycled materials, to design buildings with more durability, and to apply processes of construction with lower levels of effect [49]. Municipal solid waste products are components that interact closely with pollutants, most notably greenhouse gas emissions, which contribute to global climate change [62]. According to World Bank estimates, the majority of the average and low-income countries generate approximately 1.3 billion tons of waste annually in terms of solid waste, with solid waste estimated to reach 2.2 billion tons by 2025. [63]. In most developed countries, the common strategy for overcoming the environmental challenge is the reuse of waste and renewable resources for construction materials [62]. Waste recycling and reusing discarded materials helps to conserve renewable resources while also reducing emissions and landfilling [64,65,66]. Green and environmentally sustainable structures can be constructed by utilizing these waste products as aggregates and fibers in place of natural raw materials [67].

## 2. Importance and Significance of the Current Literature Review

The primary focus of this research is on environmentally friendly construction materials. Furthermore, this state-of-the-art study examines the influence of RFs on the MPs of CBCs. The key aim of this review is to evaluate and thoroughly examine the existing literature on the impacts of various kinds of RFs on the overall behavior of composites. The compressive, flexural and tensile strength, and the durability of RF-reinforced CBCs are analyzed. This study will provide a baseline for future studies on RF-reinforced CBCs. Researchers will benefit from the study’s comprehensive overview of RF output characteristics.

## 3. Types of Waste Fibers Used in Cement-Based Composites

**Figure 1:**
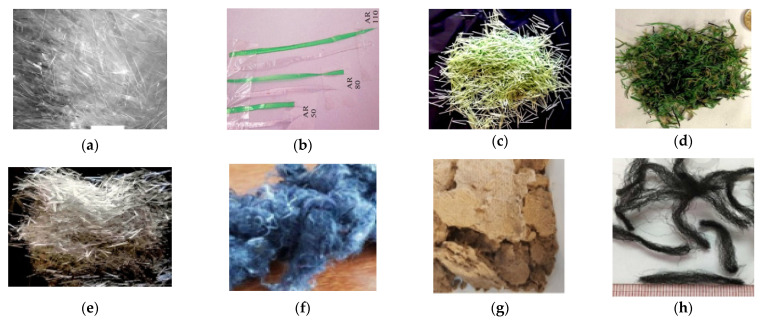

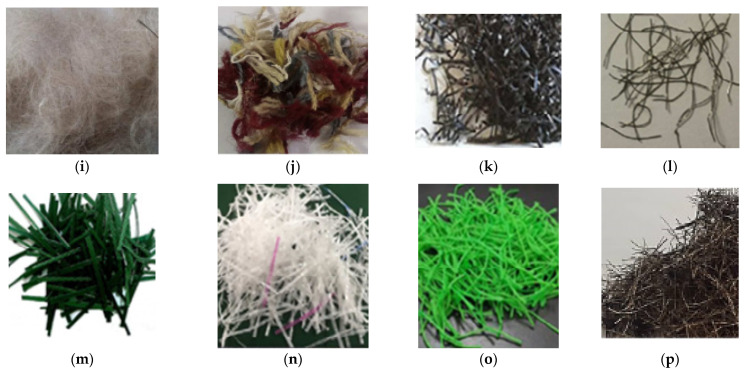
Various recycled-waste fibers used in cementitious composites. (**a**) Waste plastic fiber [74], (**b**) Waste recycled polyethylene terephthalate fiber [75], (**c**) Waste recycled PET/PE fibers from packaging [76], (**d**) Waste recycled PE/PP fibers from artificial turf [76], (**e**) Virgin PP draw-wired fibers [76], (**f**) Textile waste fiber [53], (**g**) Kraft pulp Fiber [53], (**h**) Waste polypropylene carpet fibers [77], (**i**) Waste carpet face fibers [78], (**j**) Waste carpet fibers [79], (**k**) Waste steel scrap [80], (**l**) Tire-recycled steel fibers [80], (**m**) Waste recycled plastic fiber [38], (**n**) Waste recycled woven plastic sack fiber [18], (**o**) Waste fishing-net fiber [81], (**p**) Waste recycled steel fiber [82].

The waste RFs used in CBCs are broadly classified into three categories, namely, WRPF, WRCF and WRSF. In order to produce WRPF, post-consumer plastics are processed and washed before being sliced manually through a paper shredder or a compact disk (CD) cutter device. The bottom and neck of the plastic bottles are removed for other uses [16,17,68,69,70]. Other scientists have used long plastic chips made of machined steel waste pieces in commercial vehicle plants [71]. In technological plants, plastic fibers are produced. Wasted polyethylene terephthalate (PET) bottles are used as raw materials for the replication of plastic fibers; after spinning, stretching, stabilization, winding and polygraphing, the process begins with crystallization, drying and a pneumatic conveyor, and then progresses to the dose, extrusion and filtering. The fibers have tensile forces between 263 and 550 MPa, and a specific gravity of 1.34 kg/m^3^ [72]. Some scientists used maleicon hydride grafted polypropylene to cover the surface of the WRPF for the de-bonding among the fiber and concrete. This enhances the distribution of RFs [2]. The waste carpet recycling process begins with the sorting of waste carpets according to fiber type and production, and then progresses to mechanically separating the fibers from the backing using a series of screening, shredding, cutting, tearing, screening, sifting and cleaning tools. The end product can finally meet the quality management standards [34]. A portion of the waste recycled carpet fibers used in concrete come from waste carpet recycling plants [36], [44,45,73]. The majority of WRSFs are produced from expired vehicle tires. WRSF can be separated mechanically from expired tires through shredding and cryogenic methods; it can also be produced through anaerobic thermal degradation, such as microwave-induced and conventional pyrolysis. Some of the RF used in cementitious composites are depicted in Figure 1.

## 4. Mechanical Properties of Waste Fiber-Reinforced Cement-Based Composites

### 4.1. Compressive Strength

The addition of WRSF may be advantageous for the CS of CBCs. If a higher RF content is required, extra water to address workability concerns may have a negative impact on the CS of CBCs because of an increase in porosity [20]. Comparably, the incorporation of WRSF causes ductile failure and can delay CBC failure. It was discovered that by adding high-density WRSF at a volume fraction of 5% to the concrete composite, the dry density increased, resulting in a 59% improvement in CS [83]. At volume fractions of 1 and 0.5%, a positive synergetic influence was noticed among ISF and WRSF, respectively; improved crack resistance and anchorage capability resulted in a 50% increase in CS [84]. On the other hand, the use of hybrid RF and ISF in CBC caused decreased workability and increased porosity, resulting in a decrease in CS. Concerns about the dispersion of WRSF in concrete have been addressed [20,85,86]. It was observed that when a similar kind and fraction of WRSF were used, and a traditional concrete mixer was used, the SFs were not evenly dispersed, preventing the maximum potential of WRSF from being consumed. The results indicated that using 0.26% WRSF increased the CS by 12% when a conventional concrete mixer was used; using a planetary concrete mixer resulted in an improved fiber dispersion in the matrix and enhanced the CS by 20% even at a 0.23% WRSF content. The use of a planetary vertical mixer resulted in the most homogeneous and well-dispersed SFs and an increase in the fiber content to 0.46% by volume [20]. The fiber content has a considerable effect on how concrete reacts to compressive stresses. At small fiber fractions, no significant change in the CS of concrete was observed [87]. A study demonstrated that adding a combination of ISF and WRSF up to the highest volume fraction of 0.5% did not result in a significant increase in strength, with CS only increasing from 36.69 to 37.37 MPa. In conclusion, at low proportions of WRSF, the CS of concrete is primarily determined by the internal matrix structure of the concrete, compared to SFs [21,88]. Table 1 shows various steel fibers, and their physical parameters, proportions used and influence on the MPs of CBCs.

The addition of silica fume (SF) creates a dense and compact cement matrix, and increases the bond strength among the fiber surface and the surrounding matrix, thereby improving the CS and ductility of WRSF concrete [105,106]. However, increasing the fiber content beyond the threshold value has a detrimental effect on the cement matrix structure, ultimately resulting in the fall of the concrete’s CS. This investigation was conducted using WRSF fibers with varying volume contents up to 0.75%. The results indicated a 5% increase in CS with a 0.5% fiber content, but an unfavorable impact of fiber incorporation was noted when the WRSF was 0.75% content by volume, resulting in non-uniform fiber dispersion and a non-homogeneous cement matrix; this inconsistency in the composites matrix eventually resulted in an 8% decrease in CS [95]. Similarly, 3% of WRSF by mass was recommended as the optimal dosage for higher roller-compacted concrete’s CS [107]. With a fiber volume content of 0.46%, a decrease in CS from 33.61 to 31.60 MPa was observed, and it was determined that the random distribution of WRSF in CBCs might cause fiber congestion, resulting in a small decrease in CS. [23]. Another study discovered an optimal content of hybrid WRSF and ISF. By combining 30% ISF and 70% WRSF with a total content of 1%, the CS improved by 5–10%, while a fiber fraction of 1.25% resulted in a CS loss of 5% [108]. The shape of the fibers, their surface morphology, and the quantity of rubber affixed to the WRSFs from waste tires all significantly affect the concrete’s CS. With the addition of 0.46% of rough and randomly distributed WRSF, superior resistance was observed against crack occurrence, as well as a 25% increase in the CS [92]. The existence of rubber attached to the surface of WRSF, on the other hand, has a negative impact on the concrete’s CS. The hydrophobic nature of rubber and the lack of adhesion to the surrounding matrix has an unfavorable influence on the performance of concrete [91]. The results indicated that the CS value decreased from 135.5 to 130.2 MPa, but WRSF without any rubber attached to the surface increased the CS value from 135.5 to 141.3 MPa (4.3%). The increased mechanical bonding caused by frictional stress created by the corrugated surface and the geometry of the WRSF results in a rise in CS [109]. The WRSF provides resistance to cracks through a bridging effect and lateral crack resistance, resulting in the enhanced CS of CBCs [104,110,111,112].

In general, the addition of WRPF resulted in the decreased CS of CBCs [32,41,42,71,113]. The loss of CS was documented in several studies carried out to investigate the MPs of CBCs comprising PET waste fibers of varying content and length [69,114,115,116]. When 1% PET waste fiber was incorporated into cement mortars, no CS enhancement was observed, while 1.5% PET waste fiber content reduced the CS [117]. Numerous researchers have investigated the incorporation of metalized WRPF into CBCs [32]. A study used waste fibers with varying lengths (5, 10 and 20 mm) and volume contents (0.5, 1.0, 1.5 and 2.0%). Their findings indicated that adding 1% fiber volume resulted in a slight decrease in CS; however, adding more fiber volume resulted in a greater decrease in CS. Increased the fiber length increased the CS loss. [19]. Another study examined the effect of high-density polyethylene RFs on the MPs of concrete. Fibers of 0.4, 0.75 and 1.25% content and two different sizes were used. There was no effect on the CS of concrete with the addition of the fibers [39]. Similarly, another study was performed on the MPs of concrete beams using PET waste RFs produced from bottles. Various concentrations of waste fibers ranging from 0.25 to 2.0% were used, and the results indicated a slight increase in concrete CS up to a proportion of 1% fiber. A decrease in CS was observed as the amount of fiber increased. The improvement in CS could be attributed to the fibers’ proper dispersion within the mix. Furthermore, the fibers reduced the propagation of microcracks, lengthening the time before they fail, and the samples required extra load to expand the cracks. Additionally, the reasons for the decrease in CS for more than 1% fiber addition are related to the production of bulk and the segregation of fibers [30,68]. The same results were also observed in another study [118]. It was noted that the addition of 1% PET waste fiber increased the strength of concrete by approximately 5.2 and 7.3%, when two different aspect ratios of 35 and 50 were utilized in the concrete as reinforcement, respectively [119]. Comparable results have been observed in other studies when different kinds of WRPF were used in CBCs [31,120]. Another study conducted on concrete containing straight and deformed WRPF of varying lengths and volume content reported a CS decrease of approximately 0.5–8.5% for both straight and deformed fibers. The CS loss was greater for straight fibers in a smaller quantity, whereas the deformed fiber’s negative effect was greater at higher quantities [41].

The influence on MPs of concrete incorporating distinct amounts of WRCF ranging from 0.25 to 1.25% was investigated. It was discovered that increasing the fiber content decreased the CS. However, this decrease was not excessive, and the CS remained within the permissible limit for structural applications. The results indicated that the concrete CS decreased by 2.14%, 6.14%, 10.23%, 14.8% and 20.76% at fiber fractions of 0.25%, 0.50%, 0.75%, 1.0% and 1.25%, respectively [36]. The decrease in CS was possibly due to the presence of porosity and voids within the matrix because of the WRCF’s addition and the existence of a weak bond at the fiber-matrix transitions [121]. Similarly, a further study on the creation of eco-friendly concrete containing varying proportions of WRCF was carried out. A slight decrease in the CS was found as the WRCF proportions were increased. At 91-days of curing age, the reduction range was 1.6–20.8% compared to the reference mix. While comparing the CS with the age of concrete, the 91-day CS of WRCF concrete was increased by 2.8–21.3% from 28-days, and by 9.7–23% from 7-days of age [36]. Additionally, the researchers investigated the combined influence of WRCF and palm oil fuel ash on the CS of eco-friendly concrete. It was discovered that substituting 20% palm oil fuel ash for cement and 0.5% WRCF reduced the CS by 18.2%, 16.3% and 5.4%, at 7, 28 and 91-days age, respectively, compared to the reference sample [36]. The use of WRCF showed a considerable reduction in CS by 15%, 35%, 23% and 51% at 0.5%, 1.0%, 1.5% and 2.0% fiber contents, respectively, at 1 day of curing age. This reduction was mitigated to some extent as the curing age of the concrete samples increased [35]. The same results were obtained when WRCFs of varying shapes and volume contents were used [34,45]. A slight decrease in the CS was noted when waste-recycled nylon fiber from carpets was incorporated into the CBC [122]. Furthermore, it was observed that adding 1% waste propylene carpet fibers had no discernible adverse effect on the CS of CBCs [123].

**Figure 2:**
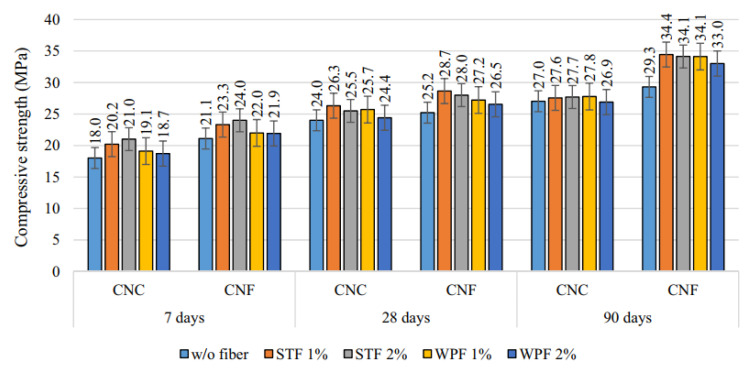
CS of control mix containing natural coarse aggregate (CNC) and control mix containing natural fine aggregate (CNF) with and without fibers [74]. Steel fiber (STF); waste plastic fiber (WPF).

**Figure 3:**
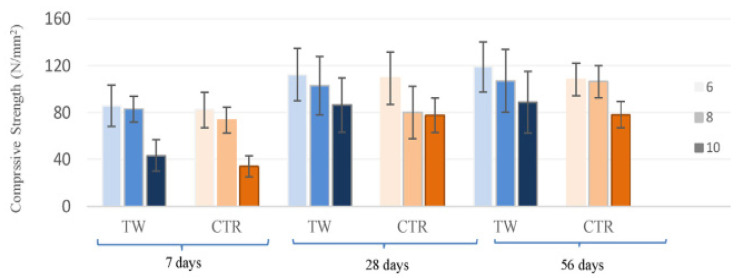
CS of composites at different ages with various recycled waste fiber additions [53]. Textile waste (TW); control kraft pulp (CTR).

In the comparison of the CS of mixes, a control mix containing natural coarse aggregate only represented by CNC, and a control mix containing natural fine aggregate only represented by CNF with and without fibers is shown in Figure 2. The water:cement ratio is 0.34 for the CNC and CNF mixes. The figure indicates that the mix CNF has a higher CS than CNC at all ages. Finer aggregates enabled a more dense pore structure and improved the interface amongst the cement matrix and aggregate. Moreover, fiber addition enhanced the CS of the mixes compared to the control mixes. This improvement was greater with the addition of SF than the waste plastic fiber due to the increased mechanical strength of SFs. Additionally, the improvement in CS was more noticeable in FRCs with age, where the CS at 90 days was 25% greater than the CS at 28 days. The increased CS of FRCs may be attributed to a strengthened fiber–matrix bond caused by continuous hydration at later ages [74]. Figure 3 represents the CS of FRCs containing two types of fibers, including textile waste (TW) and control kraft pulp (CTR) fibers at 6, 8 and 10% contents by weight of cement. TW with 6, 8 and 10% mixes with 0.42, 0.44, 0.44, 0.40, 0.5, 0.5, 0.45, 0.4 and 0.45 water:cement ratios for the ages of 7, 28 and 56 days were observed. CTR with 6, 8 and 10% mixes with 0.43, 0.44, 0.44, 0.42, 0.42, 0.35, 0.45, 0.39 and 0.35 water:cement ratios for the ages of 7, 28 and 56 days were observed. The CS decreased significantly as the fiber content increased with each type of fiber. The TW fiber composite with 6% fiber content had the highest CS, between 85.8 and 119.1 N/mm^2^ at 7 and 56 days. The composite with 10% TW fibers had the lowest values, between 43.2 and 88.9 N/mm^2^ at 7 and 56 days. This decrease may be explained by the fact that increasing the fiber fraction results in an increase in voids, which weakens the material [53].

**Figure 4:**
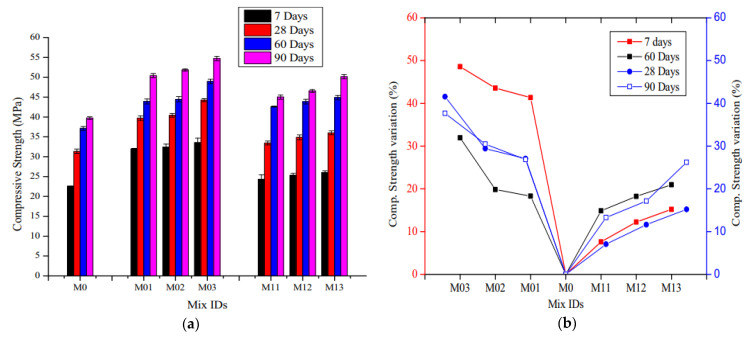
(**a**) CS at curing ages of 7, 28, 60 and 90 days; (**b**) percentage variation in CS at curing ages of 7, 28, 60 and 90 days [124]. The mixes M0, M01, M02 and M03 represent self-consolidating concretes with MSF contents of 0, 0.5, 1.0 and 1.5 %, respectively. The mixes M11, M12 and M13 represent self-consolidating concrete with WRSF contents of 0.5, 1.0 and 1.5 %, respectively.

**Figure 5:**
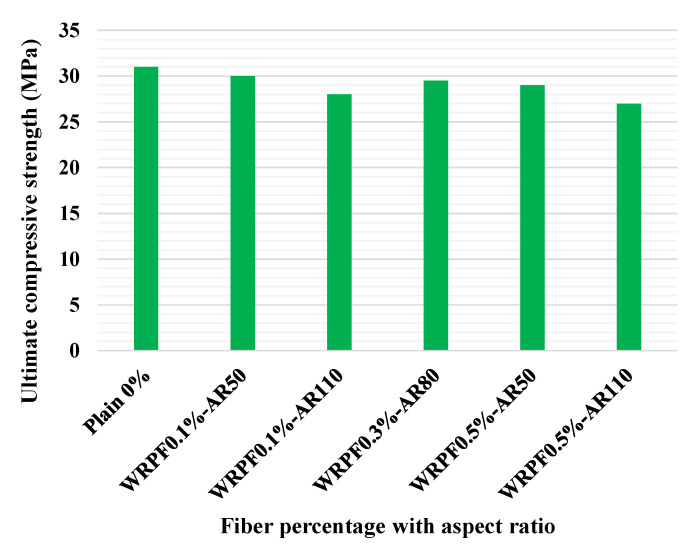
Ultimate CS of the control concrete and Waste recycled plastic fiber (WRPF) concrete with various fiber aspect ratios [75]. Waste recycled plastic fiber (WRPF); aspect ratio (AR).

The effect of manufactured steel fiber (MSF) and WRSF on the CS of SCC is displayed in Figure 4. The mixes M0, M01, M02 and M03 represent self-consolidating concretes with MSF contents of 0, 0.5, 1.0 and 1.5 %, respectively. The mixes M11, M12 and M13 represent self-consolidating concrete with WRSF contents of 0.5, 1.0 and 1.5 %, respectively. Figure 4a indicates that the 7-day CS of WRSF has no substantial variation in comparison with the control mix (M0). However, at the later ages, i.e., 28, 60, and 90 days, there was a significant improvement in the CS of self-consolidating concrete with the addition of WRSF (M11, M12, M13) compared to the control mix, which was minutely smaller than the self-consolidating concrete with micro–SF. Figure 4b depicts the percentage variation in the CS of fiber WRSF composites with respect to the control mix. A CS increase of 37.68% was observed at 1.5% content by volume of MSF after 90 days, in comparison with the control mix. The enhancement in CS with WRSF at 1.5% content by volume was only 26.22% after 90-days. The CS was found to be relatively equivalent at 60 days for 0.5 and 1% MSF and WRSF in self-consolidating concrete, respectively. However, when specimens were investigated for their compressive performance, it was discovered that WRSF was more efficient than MSF at resisting cracks and delaying a smooth failure of specimens devoid of high damage, i.e., the broken matrix remained attached to the WRSF [124]. Figure 5 depicts the ultimate CS of the control concrete and WRPF reinforced concrete. Whereas the ultimate CS of concrete means its maximum compressive strength, the CS of the control concrete was 30.8 MPa, which is 6% higher than the average of all of the WRPF reinforced concrete samples. Additionally, the variation in CS of WRPF reinforced concrete compared to the control concrete was greater at higher fiber contents than the lower fiber content. Moreover, the reduction in CS was related to the aspect ratio of the WRPF fiber; samples with longer fibers performed worse than those with shorter lengths. Considering the results in Figure 5 and their variation intervals, it could be concluded that the addition of fibers to concrete does not significantly affect its compressive strength [75].

### 4.2. Flexural Strength

Numerous research studies found an increase in FS when WRPF was added to the concrete. A study was conducted to determine the effect of waste high-density polyethylene fibers on the MPs of concrete. Two distinct sizes of fiber and content in the range from 0.4 to 1.25% were utilized. The results revealed a steady rise in the FS of the CBCs. The specimens with smaller fiber lengths and diameters resulted in a greater FS than the specimens with longer fibers and higher diameters [39]. Similar results were observed in numerous studies on cementitious composites containing distinct fiber quantities and sizes [31,38,40,125]. The effect of WRPF addition on the MPs of cement mortar was examined. The improvement in FS was observed at a 30% volume content after 28 days, and at a 50% volume content after 63 days. The factors contributing to the increase in the FS of the concrete composites are the presence of fibers in the concrete tension zone, which resist tensile stresses and microcracks for a small period of time, thereby increasing the microcrack bridging action [125]. Several studies reported that the maximum FS improvement achievable with WRPF-reinforced concrete is at 0.5% fiber content in CBCs [32,33]. Another study reported FS improved by 19 and 7% when the fiber proportions were 0.5% and 1.0%, respectively. On the other hand, metalized WRPF was added to the green CBCs to investigate its effect on the concrete’s various properties. It was found that an increasing amount of fiber in the concrete composite resulted in an increase in FS, with the maximum value observed at 0.5% fiber content. Additionally, increasing the fiber content to 0.75% resulted in a decrease in the concrete FS, but it remained higher than the control mix [32,33,37]. Similarly, another study reported that WRPF reinforced concrete with a 0.4% fiber dosage exhibited the highest FS enhancement. At the age of 28 days, concrete samples with a waste fiber content of 0.2 and 0.4% had an improved FS of 23.8 and 35.6%, respectively, compared to the reference sample. Increased fiber dosages resulted in a decrease in concrete strength. Additionally, the presence of waste fibers may act as a barrier to crack growth, and may move across the cracks to transfer interior forces, increasing FS. [113]. Some authors described the effect of crimped and smooth WRPF on the properties of cement-based materials. The samples with crimped fibers exhibited better strength than the samples with smooth fibers [115]. The results of an experimental study revealed that, with fiber content increments, a slight decrease in FS was noticed for different fiber sizes. Furthermore, a 9% reduction in FS was observed as an average value between all of the WRPF-reinforced concrete samples compared to the samples of plain concrete (PC). The same results were also observed by other researchers [19,42]. At a curing age of 28 days, the FS was reduced by 4.6%, 7.2% and 12.4% when WRPF was used proportions of 0.5%, 1.0% and 1.5% in concrete, respectively. Additionally, some researchers reported a decrease in FS when various lengths and volume contents of WRPF were incorporated [43], [126].

The FS might decrease due to the improper pouring and placement of concrete, which results in the formation of voids and pores within the matrix [127,128]. Different volume contents of WRCF with identical fiber sizes were incorporated. It was observed that increasing the fiber amount improved the FS to a certain extent. At 28 days of age, the FS of the FRCs were between 3.64 and 4.11 MPa, with 0.70% fiber content having the highest FS. The enhancement was more significant than that of the PC specimen [129]. Likewise, the addition of WRCF influences the MPs of CBCs. The volume content of WRCF fiber was varied between 0.5 and 2.0%, with a 0.45 mm fiber diameter and a 30 mm fiber length. The results indicated an increase in FS due to the addition of fibers. At 1 day of curing age, all of the specimens containing fibers had a greater FS value than the PC specimens. On the other hand, at 7 and 28 days of curing age, a slight reduction in FS at 1.0% of fiber content was noted. At 28 days, the maximum FS was 6.25 MPa with a 0.50% fiber content. The strength was 17.9% greater than the PC, whereas the FS was reduced by approximately 17% as a result of the addition of 2.0% WRCF [35]. It has been reported that the addition of WRCF up to 1% content improved the FS of CBCs. Furthermore, another study on the manufacture of ecofriendly concrete with a WRCF of 0.25–1.25% and a length and diameter of 20 mm and 0.45 mm was carried out. It was discovered that incorporating RFs into concrete at 0.25, 0.50, 0.75, 1.0 and 1.25% proportions increased the FS by 11.23, 24.7, 20.22, 11.23 and 10.11%, respectively, at 28 days, in comparison with the plain control mix. A fiber content of 0.50% was optimal for the maximum FS. The increase in FS was due to the crack-arresting process of the fibers [36]. A similar improvement in FS in CBCs with the addition of RF was also stated by other researchers [78,79,123].

**Figure 6:**
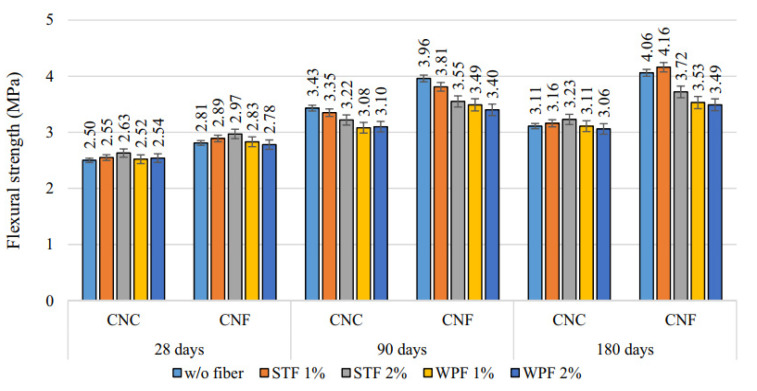
FS of control mix containing natural coarse aggregate (CNC) and control mix containing natural fine aggregate (CNF) with and without fiber [74]. Steel fiber (STF); waste plastic fiber (WPF).

Figure 6 displays the FS of mixes of CNC and CNF with and without fibers. The water:cement ratio is 0.34 for CNC and CNF mixes. The denser and compact microstructure of CNF mixes resulted in a superior FS compared to the CNC mixes. The CNF mixes exhibited around 15% improved FS compared to the CNC mixes. Mostly, incorporating fibers as reinforcement in the mixes resulted in a lower FS than the control mix, but in a few cases, a slight rise in FS was noticed. The decrease in the FS of plastic fiber-reinforced mixes was more pronounced than that of SF-reinforced mixes, particularly in CNF mixes, which is due to the lower modulus of plastic fiber elasticity compared to the SFs [74].

**Figure 7:**
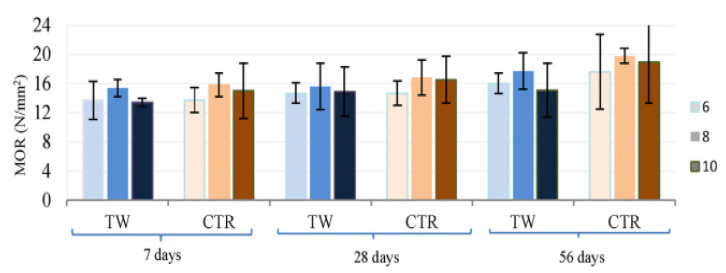
Moduli of rupture of the composites at different ages with various recycled waste fiber additions [53]. TW: textile waste; CTR: control kraft pulp.

Figure 7 represents the FS of FRCs containing two kinds of fibers, including TW and CTR fibers at 6, 8, and 10% contents by weight of cement. TW with 6, 8 and 10% mixes with 0.42, 0.44, 0.44, 0.40, 0.5, 0.5, 0.45, 0.4 and 0.45 water:cement ratios for the ages of 7, 28 and 56 days were observed. CTR with 6, 8 and 10% mixes with 0.43, 0.44, 0.44, 0.42, 0.42, 0.35, 0.45, 0.39 and 0.35 water:cement ratios for the ages of 7, 28 and 56 days were also observed. The TW of 6% and 10% content by mass of cement indicated approximately similar moduli of rupture (MOR) at all curing ages, while 8% content showed about 15, 6, and 17% improved MOR at the curing ages of 7, 28 and 56 days, respectively. However, the CTR samples showed a variance in MOR of less than 5% for different proportions of fibers added. An average MOR of 17.7 N/mm^2^ of TW fiber-reinforced samples was less than the MOR of CRT samples of 19.8 N/mm^2^, which was 9% for an 8% content of both fibers at 56 days. Therefore, a fiber content of 8% was found to be optimal for both fibers in terms of MOR. Additionally, an increase in the MOR was observed as the composite aged from 7 to 56 days; for TW, the increase was 15%, and for CTR, the increase was 26% [53].

**Figure 8:**
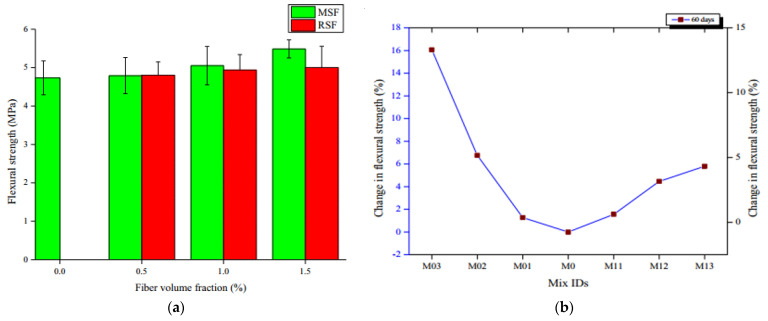
(**a**) FS of composites at 60 days of age [124]; (**b**) percentage variation in FS at 60 days of age [124]. The mixes M0, M01, M02 and M03 represent self-consolidating concretes with MSF contents of 0, 0.5, 1.0 and 1.5 %, respectively. The mixes M11, M12 and M13 represent self-consolidating concrete with WRSF contents of 0.5, 1.0 and 1.5 %, respectively.

**Figure 9:**
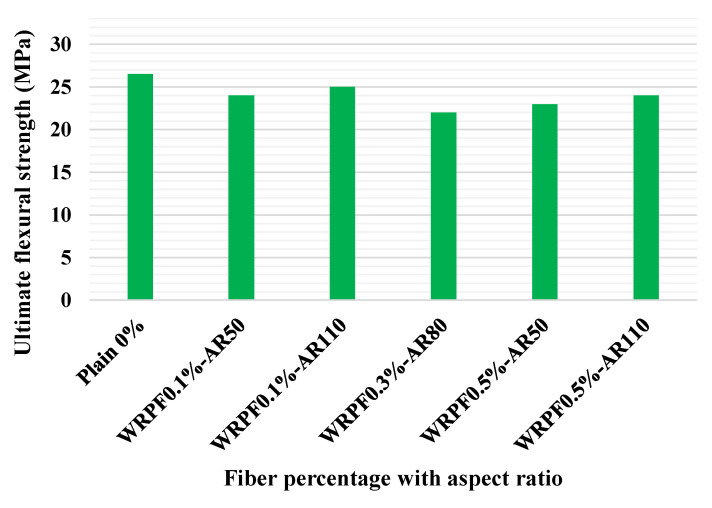
Ultimate FS of the control samples and Waste recycled plastic fiber (WRPFs) concretes with various fiber aspect ratios [75]. Waste recycled plastic fiber (WRPF); aspect ratio (AR).

Figure 8 depicts the FS of the control self-consolidating concrete and self-consolidating concretes containing MSF and WRSF in different proportions at the age of 60 days. No major enhancement in the FS was observed for self-consolidating concrete with WRSF compared to the control self-consolidating concrete, except for the self-consolidating concrete with 1.5% MSF addition, as shown in Figure 8a. Furthermore, in Figure 8b. The mixes M0, M01, M02 and M03 represent self-consolidating concrete with MSF contents of 0, 0.5, 1.0 and 1.5 %, respectively. The mixes M11, M12, M13 represent self-consolidating concrete with WRSF contents of 0.5, 1.0 and 1.5 %, respectively. It was noticed that the extreme improvement was 5.79% in the FS of self-consolidating concrete with the addition of WRSF, while a 16% improvement was noted in FS with the addition of MSF. Hence, the incorporation of fibers represented an improvement in FS with a higher volume fraction of fibers [124]. Figure 9 shows the ultimate FS of normal specimens and specimens containing WRPF with various fiber aspect ratios. The ultimate FS of concrete means its maximum flexural strength. It was noted that WRPF-reinforced specimens have comparable FS to the normal specimen, with a difference of only 7% [75].

### 4.3. Splitting Tensile Strength

Over the last few years, extensive research has been performed on the MPs of WRSF composites [96,130,131,132,133]. The research showed that the incorporation of 0.75% volume content of WRSF and industrial SF in concrete increased the STS by 28% and 26.33%, respectively, compared to the PC [95,134]. Inconstant fiber diameters and lengths appeared to be responsible for an extra interlocking mechanism, as a 0.75% volume content of WRSF increased STS by 50% over the PC [135]. SFs lower than the optimum fraction have a negative influence on the STS of concrete by introducing congestion into the composite, even though adding SFs near the optimum fraction reduces the porosity and improves the performance of the composites [23,98]. This phenomenon was detected when 0.4% WRSF by volume was added, causing a reduction in the STS of concrete. Likewise, a considerable reduction in load-bearing capability was noted when the main reinforcement was substituted with WRSF [136]. Thus, a 14% increase in STS was observed when the fiber volume content increased to 0.6% [98]. An effort was made to improve the MPs of SCC by adding WRSF, ISF and WRPF in a ratio of 1.5% by volume. Compared to the reference sample, WRSF increased the STS by 25%, whereas the hybrid combination with WRSF 0.5% and ISF 1% exhibited the greatest improvement in STS. It was realized that the increased mechanical anchorage and effectiveness of WRSF and ISF in bridging cracks enabled the strength to increase [85,110]. According to the desirability function analysis, the optimal amount of WRSF in concrete reinforced with a mono-fiber is 1.5%; however, a hybrid combination of WRSF 0.5% and ISF 1% resulted in the superior MPs of the composite [84]. The outcome of a desirability function analysis depends entirely on the features considered. By incorporating a single additional factor, namely global warming potential, the optimum volume fraction of WRSF and IDF became 1.35% and 0.15%, respectively [90]. STS increased gradually with the addition of 0–1% WRSF with 0.25% palm oil fuel ash. The STS increased by 22.85% with 1% WRSF and 15% palm oil fuel ash. It was determined that the WRSF addition protected from the formation of internal fractures [101,102,137]. The same trend was observed for the STS of composites for roadway structures with the addition of WRSF with a 0.5% higher specific gravity [138]. With the addition of 0.5% WRSF, the concrete STS increased by 43% when compared to the control mix, while composites with ISF of 0.25% volume content demonstrated a 9% increase in STS [104]. Additionally, it was discovered that rubber fragments affixed to the surface of WRSF act synergistically to increase the concrete’s tensile capability. The fibers remained unharmed through the burning process to eliminate the rubber fragments, and the STS was increased by 13% with the addition of 1% WRSF and 40% SF [105].

On the other hand, several studies on the use of WRSF in concrete STS have reported inconsistent results [139,140,141,142,143]. Rubber particles attached to WRSF were intended to have a negative influence on the STS of concrete. Rubber is a soft material by nature, as opposed to the dense matrix of cement, which resulted in an elastic inequity and acts as voids due to the minor resistance to the load [139,141]. The rubberized concrete with WRSF was investigated, and it was noted that the STS of the concrete decreased by 50% as a result of the addition of rubber, although the WRSF addition was beneficial in achieving strength, and it reduced the strength loss of the composite [144]. Similarly, the improvement of STS was noticed in recycled aggregate concrete with a 3% addition of WRSF [145]. The influence of WRSF extracted from various tire scraps with different aspect ratios on the concrete STS was examined. No major enhancement in STS was noticed by adding WRSF [26]. Moreover, WRSF was at risk of corrosion in the high chloride environment, which reduced the fibers’ mechanical strength; as a result, the STS of the concrete decreased [146]. A study was conducted on high-strength concrete beams with waste PET plastic bottle fibers of varying lengths and volume contents. The STS of the concrete was reduced with increasing the fiber amounts, and the lowest drop in strength was nearly 3.67% compared to the control sample when different hybrid 40 mm long and 20 mm short fibers were used, with a total fiber content of 0.75%. Thus, it was stated that 0.75% fiber content is the optimal ratio [147]. The researcher observed similar results when varying the volume content of WRPF; these findings may indicate a weakness in the fiber–cement matrix interface [42]. Similarly, a reduction in the STS of cement mortars was noted with varying volume contents of WRPF [69]. Additionally, a concrete mixture containing 0.4, 0.75 and 1.2% WRPF was investigated. Two types of fibers were used in accordance with the aspect ratio. The outcome demonstrated the enrichment of STS by incorporating fibers into the concrete. The 0.4% fiber content was determined to be optimal for the maximum STS. Additionally, it was stated that varying the aspect ratio has little effect on the STS [39]. Similarly, a study was conducted to determine the effect of WRPF on the MPs of conventional concrete and binary cement concrete [148]. Furthermore, a study was conducted on cement mortars utilizing a range of waste PET fiber volume fractions. The results indicated that the addition of 0.5% fibers increased the STS to 16% [68]. Additionally, the ability of the novel concrete composite to provide resistance against the stresses of tensile forces was observed [5]. Additionally, other studies reported that the STS improved as the fiber length and aspect ratio increased. Further investigation revealed that adding 0.5% and 1.0% of type C WRPF to concrete increased its STS by nearly 21% and 33%, respectively. The STS enhancement of fiber types A and B was addressed sequentially in comparison to fiber type C [19,114]. Enhancements in the STS of 15.5% and 24.9% were noticed with the addition of 1.0% of PET fibers with varying aspect ratios of 35 and 50, respectively [119].

According to several scholars, the STS improvement of concrete with WRPF was greater with the addition of fiber percentage contents ranging from 0.25% to 2.0% by volume [30,31]. A 12.5% increase in STS was observed in comparison to the PC when 1% waste recycled PET fibers were used. Additionally, another study reported that concrete with WRPF had the highest STS increment with a fiber content of 0.5% [37,118]. Moreover, the highest STS enhancement was observed when a 0.75% volume of WRPF was added to the concrete [32,33]. It was observed that when the WRPF content was increased in the composites, the STS improved significantly. Increases of 16.5%, 24%, 25.5%, 19% and 14.4% were noticed with WRPF fiber contents of 0.25%, 0.50%, 0.75%, 1.0% and 1.25%, respectively, compared to the normal concrete. The optimal WRPF content was determined to be 0.75%. This effect could be attributed to the increased interaction between WRPF and the cement matrix [32,149]. Moreover, the presence of fibers in the concrete enhanced the barrier to oppose to indirect tension, increased the strain capacity of concrete, and resulted in a greater STS [150,151]. A study was conducted on the MPs of concrete using WRCF. It was observed that increasing the fiber content improved the STS of concrete at various curing ages. Increasing the fiber content by 0.5%, 1.0%, 1.5% and 2.0% resulted in an increase in STS of 9%, 18.2%, 27.3% and 15.2% after 1 day of curing. Additionally, it was found that the highest increase in STS appeared when the volume content of fiber was 1.5% of the concrete [35]. It was reported that specimens of green concrete containing WRPF performed significantly better than the control mix. At 28 days of age, the different fiber volume fractions of 0.25%, 0.50%, 0.75%, 1.0% and 1.25% improved the STS by 17.2%, 26.2%, 20.3% and 17.2%, respectively, compared to the control mix. The optimal fiber content for the STS was 0.50% [45]. Similarly, the addition of WRCF enhanced the STS of CBCs. The effect of fiber bridging on the splitting portions of the samples acts as a stress transfer from the constituents of the concrete to the fibers, which is why it sustained the total splitting tensile stresses gradually and eventually improved the STS of the samples [36]. Moreover, at 180 days of age, an increase in STS of 15.4%, 17.9%, 19.2%, 11.55% and 7.7% was observed with fiber fractions of 0.25%, 0.50%, 0.75%, 1.0% and 1.25%, although this strength was slightly greater than that from 91 days of age. Furthermore, the greatest STS increase was observed at the fiber proportions of 0.75% and 0.50%, at 180 and 91 days, respectively [36]. Similar outcomes for enhancements in STS were also stated in numerous studies [127,128,150,152].

**Figure 10:**
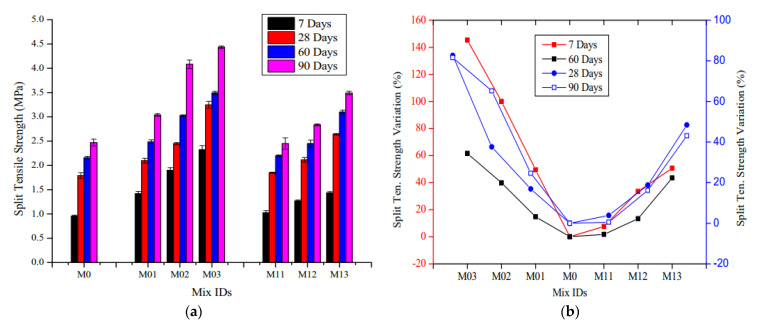
(**a**) STS of concrete at different ages [124]; (**b**) percentage variation in the STS of concrete at different ages [124]. The mixes M0, M01, M02 and M03 represent self-consolidating concretes with MSF contents of 0, 0.5, 1.0 and 1.5 %, respectively. The mixes M11, M12 and M13 represent self-consolidating concrete with WRSF contents of 0.5, 1.0 and 1.5 %, respectively.

**Figure 11:**
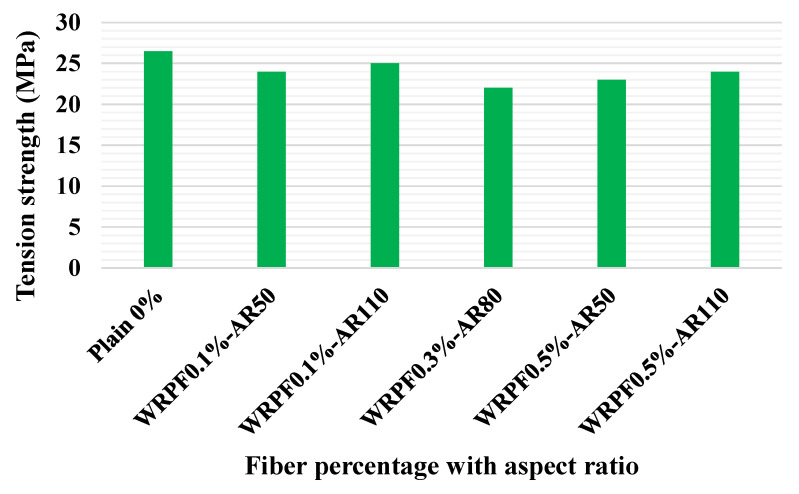
Ultimate STS of control samples and WRPFs concrete with various fiber aspect ratios [75]. Waste recycled plastic fiber (WRPF); aspect ratio (AR).

The influence of MSF and WRSF on STS of SCC is shown in Figure 10. The mixed M0, M01, M02 and M03 represent self-consolidating concrete with MSF contents of 0, 0.5, 1.0 and 1.5% respectively. The mixes M11, M12 and M13 represent self-consolidating concrete with WRSF contents of 0.5, 1.0 and 1.5 %, respectively. Figure 10a indicates that a rise in STS was recorded at 7 days with WRSF when compared with the normal SCC, which further improved at later ages. From Figure 10b, a significant rise in STS can be observed with the addition of MSF fiber. A maximum of 50% improvement can be seen with the addition of WRSF at 7 days with 1.5% fiber content (M13), which was further improved at later ages. When compared to plain SCC without fibers, WRSF and MSF exhibited superior tensile behavior at later ages [124]. Figure 11 shows that the inclusion of WRPF with various fiber aspect ratios resulted in a drop in the STS ranges from 9 to 16% when compared to the normal concrete [75].

## 5. Durability Performance of Waste Fibers in Cement-Based Composites

Increased porosity was observed in reinforced concrete with WRSF, and the ultra-sonic pulse velocity results showed that the addition of 2% WRSF volume content reduced the ultra-sonic pulse velocity by 3–7% [130]. Due to its finer particle size, silica nano-powder acted as a filler and reduced the porosity of the cement matrix, ultimately enhancing the mechanical properties of concrete containing WRSF [83]. WRSF-reinforced concrete reduced the ultra-sonic pulse velocity by 10%, and that of hybrid fiber reinforced concrete containing ISF and WRSF was reduced by 15%. This reduction was due to the lesser concrete compaction of the WRSF mix [90]. The incorporation of WRSF in the composite caused an enhancement in impact resistance, crack resistance and shrinkage behavior [27,153,154,155,156]. Diffusion, permeation, and capillary transport are the three primary modes of corrosive agents’ ingress into concrete. The control of the width of the crack occurrence caused a reduction in the ingress of damaging chemicals into the matrix of concrete; this effect led to an overall drop in the weakening of composites and the corrosion of steel fibers. By restricting the width of the crack to 0.3 mm, a just-visible negative effect was caused by the fiber surface being damaged in the corrosive atmosphere [157,158,159]. Carbon dioxide is observed to be susceptible to corrosion in a chloride-rich atmosphere, resulting in the corrosion of SFs and a reduction in the durability of SF-reinforced concrete [146,160]. However, corrosion was discovered to considerably change the cement matrix and fiber content. WRSF treated at 350 °C converted the austenite retained in the microstructure to bainite, thereby increasing the strength of the WRSF. The electrochemical findings indicated that the WRSF was 90% susceptible to corrosion in a 3.5% NaCl solution by weight, and WRSF was observed to be more susceptible to corrosion than ISF. Similarly, following dry and wet cycles of chloride exposure, no substantial decrease in the performance of WRSF-reinforced concrete was noted, nor was any surface damage observed. Additionally, the incorporated SFs were found to be undamaged [157,161]. It was reported that new SFs and WRSF increased the durability of concrete [162]. Moreover, the less-compressed rubber particles resulted in increased water absorption and porosity, and increased the surface scaling and mortar evaporation due to freeze–thaw cycling [163]. Additionally, the influence of WRPF at 1% volume content after 30, 60, 90 and 120 days of exposure to calcium chloride, sulfuric acid, salt, alkali and sodium sulphate atmospheres indicated that sodium sulphate and alkalis have a negligible effect on the strength loss when compared to sulfuric acid, with a significant loss in strength of approximately 24% at 120 days. On the other hand, the effect of calcium chloride and salt on the reduction of CS in cementitious mixes was negligible. In general, WRPF fibers exhibited tremendous chemical resistance to salt, sodium sulphate, calcium chloride and the ambient environment [164].

**Figure 12:**
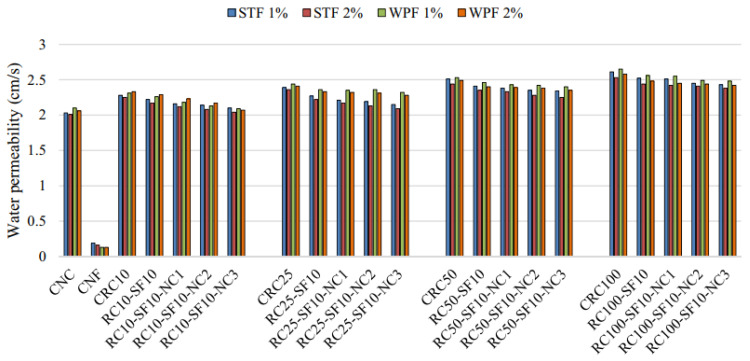
Influence of fiber content and type on the permeability to water [74]. STF: steel fiber; WPF: waste plastic fiber.

A similar trend was noticed in fiber-reinforced concrete with different types of fibers, as presented in Figure 12. There was no effect on water permeability with the addition of fibers. The recycled coarse aggregate composites showed the higher permeability to water; with an increment of recycled coarse aggregate, water permeability increased. Furthermore, a combination of SF and nanoclay in the concrete led to a gradual reduction in the water permeability of the composites [74]. Another study also reported that incorporating fibers had a negligible effect on concrete permeability [165]. Due to their volume content and type, the added fibers had no substantial impact on the water permeability of the pervious mixture of concrete [166].

## 6. Environmental Impact with the Use of Waste Fibers in Cement-Based Composites

The environmental impact of waste RF used in cementitious materials is shown in Table 2.

## 7. Discussion on the Challenges and Future Work

Based on the existing literature on WRSF-reinforced CBCs, several current issues and possible future trends can be discussed. Although tires are one of the highest ample waste materials for SF recovery, the inconsistency in their recycling procedures makes it hard to attain a regular geometry in recycled SFs and achieve a homogeneous concrete blend with evenly distributed SFs. There is a need to develop standards to obtain the finest feasible WRSF and to eradicate concerns associated with the inconsistency of the available WRSF geometries. The sustainable impact analysis of different recycling techniques must be conducted in order to determine the best cost-effective and sustainable method for recycling regular and high-quality SFs. There is a dearth of data on the properties of WRSF. The work could be expanded to include a more detailed examination of the qualitative and quantitative analysis of WRSF reprocessed from various sources.

Furthermore, bulk density and porosity are directly related to the compaction of fresh FRC and rubber affixed to the surface of recycled SFs from tires. As a result, there is a requirement to specify the specifications for the recycling of SFs from tires, and recycling factories should follow them. Additionally, WRSFs should be sorted by diameter and length prior to their application in CBCs. According to the literature, a fiber size of 0.15–0.26 mm (diameter) and 25–40 mm long fibers are preferred, as these parameters have been shown to be promising for enhancing the various MPs of WRSF-reinforced CBCs.

The CS of WRSF-reinforced composites was found to be inconsistent and depends on the fiber content, optimal dosage and fiber geometry. CS was observed to be proportional to the quantity of fibers, instead of their geometry. In general, increasing the WRSF content was observed to be favorable for improving the CS, but inconsistent outcomes have been found in past studies, necessitating additional research in the future to fully understand the effect of WRSF on the CS of concrete. The addition of secondary cementitious materials to WRSF is beneficial for increasing the CS of composites. A lower fiber content does not provide matrix homogeneity, and only the cement matrix performs its function under compressive load.

The literature contains relatively consistent data on the STS of CBCs when WRSF is added. It was stated that WRSF can convert the brittle breaking of samples to ductility, and can enhance the concrete’s STS. In order to enhance the tensile behavior of concrete, the optimal content of fibers needs to be added. The addition of less WRSF than the optimum content does not provide a sufficient reinforcing effect, whereas fiber contents greater than the optimum value have a detrimental effect on the WRSF dispersion into the mix, resulting in increased porosity and weak points in the matrix, which eventually influence the MPs of CBCs. Though the optimum content of WRSF is critical, no definite conclusion about the optimal content of WRSF can be drawn due to the variability of the results.

Flexural and tensile strength is influenced by fiber geometry, with longer fibers providing a more effective reinforcing mechanism and increasing the concrete’s flexural and tensile strength. The quantity of rubber affixed to the WRSF from tires and heat treatment are highly dependent on one another. Minimal heat in recycling is ineffective at removing the rubber from the fibers, whereas excessive heat can damage the WRSF. As a result, caution should be exercised during the treatment of SFs with heat recycled from waste tires in order to ensure that any rubber particles that adhere to the surface are removed. Any deterioration in the MPs of the fibers affects the properties of the resulting composite.

## 8. Conclusions

The research assessed in this study demonstrated that when waste recycled steel fiber (WRSF) is utilized in cement-based composites (CBCs) under optimal conditions, it can provide equivalent mechanical properties (MPs) to industrial steel fiber (ISF) without impairing the workability significantly. Thus, the utilization of WRSF in the construction sector may provide economical and sustainable composites that possess adequate crack resisting ability and enhanced MPs. Additionally, recycling steel fibers (SFs) from waste tires creates a new revenue stream for reusing expired and waste tires, which is more eco-friendly, provides cost-effective energy, and reduces mosquito-propagating places that circulate deadly infections like dengue fever and malaria.

Recent advances in WRSF-reinforced concrete, as well as its MPs and durability, were discussed in this study. The concluding remarks include the fact that recycling SFs from waste tires is not only environmentally friendly but also a cost-effective method of generating energy during the cement manufacturing process. The incorporation of hybrid fibers, i.e., a combination of WRSF and ISF in CBCs, provides a more robust mechanism for structural loadings. In accordance with ISF, WRSF-reinforced CBCs can provide comparable performance under flexural loading.

The addition of recycled-waste fibers (RFs) has no discernible effect on the water permeability of CBCs. The corrosion of WRSF was observed in chloride-rich environments, resulting in the weakening and deprivation of matrix interaction and eventually a reduction in the composite’s performance. Additionally, WRSF was more corrosive than ISF, which could result from WRSF’s longer use than ISF. Likewise, rubber fragments affixed to the surface have no effect on corrosion, which may be due to rubber’s obstructive impact on water and other chlorides.

The research interest in WRF-reinforced CBCs has increased significantly over the last few years, and the subject is primarily approached experimentally to determine the composite’s mechanical properties. There is a particular emphasis on the utilization of recycled construction materials, with plastic and metals being the most extensively studied. Additionally, the significance of the construction industry’s transition of concrete to sustainability is extensively recognized. There is a disparity in investing in the potential of waste RF-reinforced composites in addressing its triple bottom line. Accelerated aging conditions were applied to textile waste fibers and reference composites. The results for the textile waste FRCs demonstrated a significant improvement in their mechanical performance (at least 10%) over the reference samples.

The experimental tests, including the workability and compressive, splitting tensile, and flexural behavior, exhibited that incorporating waste recycled plastic fibers (WRPFs) in CBCs produces residual strength capacity, with a scarce effect on its volumetric weight and ultimate flexural and compressive strength. The study supports the use of concrete with WRPF by understanding its behavior, which could help to understand the mechanical strength of fiber-reinforced concrete structures with WRPFs.

The effects of waste RFs on the slump, compressive, splitting-tensile, and flexural strength, energy absorption, ductility and durability of concrete were reviewed. A huge number of publications were collected and examined for this purpose. The current study prompts the building sector to adopt a new type of concrete made from various RFs recovered from waste plastics, carpets and steel. Further research on the influence of fiber orientation during placement is possible. For the better applicability and suitability of this material, the fiber orientation ratio, and top and bottom alignment angles of the fibers could be examined. SFs collected from discarded tires in concrete could be a beneficial material in the building sector.

## Figures and Tables

**Table 1:** Mechanical properties of the different waste recycled steel fiber (WRSF) used in cement-based composites.

Fiber Name	Fiber Length (mm)	Fiber Diameter (mm)	Aspect Ratio	Content (%)	Compressive Strength (%)	Flexural Strength (%)	Tensile Strength (%)	Reference
Recycled steel fibers	20	0.15	133	20	−11.6	22.7	45.16	[89]
	50	0.15	333	1.5	40	25	-	[90]
	55	0.125	440	1.5	−9.3	40.5	-	[91]
	31.4	0.24	131	0.46	25.45	−15	-	[92]
	22	0.2	110	6	-	23.07	-	[93]
	16.5	-	-	1	0	-	−21.4	[94]
	50	1.2	42	0.75	−8	40	28	[95]
	35	1	35	2.4	13.9	-	35.9	[96]
	40	0.15	267	0.75	18	25	30	[97]
	50	0.6	83	1.6	8.6	67.85	32.3	[26]
	20	0.18	111	0.6	-	-	14	[98]
	25	0.26	96	2	23.3	55.27	-	[99]
	35	0.2	175	10	0.66	−7	-	[100]
	25.4	0.25	102	1	12.5	31.27	22.85	[101]
	60	0.27	222	4	26.7	-	78.6	[102]
	13.94	0.25	56	0.46	−3	-	−10	[23]
	208	2	104	4	1.8	-	172.8	[103]
	26.17	0.25	105	2	22.2	30	42.8	[104]
	26	0.258	101	0.23	19.95	15.87	-	[20]

**Table 2:** Different recycled-waste fibers used in cement-based composites.

Fiber Type	Recycling Source	Concrete Type	Impact on Sustainability	References
Polyethylene terephthalate (PET)	Bottles	Concrete	Yes	[167]
		Concrete	Yes	[168]
		Fiber Reinforced Concrete (FRC)	-	[169]
			-	[170]
			-	[41]
		Plastic Fiber Reinforced Concrete (PFRC)	Yes	[171]
		Ring-shaped PET (RPET) fiber in concrete	Yes	[172]
	-	Neat asphalt concrete mixture	Yes	[173]
	-	Fiber Reinforced Concrete (FRC)	-	[174]
Plastic	Bottles	Light weight aggregate concrete	Yes	[175]
	Doors	Waste plastic fiber reinforced concrete	-	[176]
	Plastic bags	Self-compacting concrete (SCC)	-	[177]
Waste plastic fibers	Beverage bottles		-	[17]
Plastic	-	Concrete	Yes	[178]
	-	Fiber Reinforced Concrete (FRC)	Yes	[179]
	-	Self-compacting concrete (SCC)	-	[180]
	-	Concrete	Yes	[181]
Waste plastic	-		Yes	[182]
Glass fiber reinforced plastic (GRRPF) waste	-		Yes	[183]
Polypropylene (PP) carpets	Textile	Fiber Reinforced Concrete (FRC)	Yes	[44]
	Textile/ agriculture	Concrete	Yes	[184]
	Agriculture Textile	Concrete	Yes	[185]
	Waste carpet	Fiber Reinforced Concrete (FRC)	-	[45]
Polythene	Domestic waste plastic	Fiber Reinforced Self Compacting Concrete (FRSCC)	Yes	[186]
		Fiber Reinforced Concrete (FRC)	-	[187]
Steel	Tires	Reactive powder concrete (RPC)	-	[188]
		Reinforced concrete (RC)	Yes	[108]
		Two-Stage Concrete	Yes	[189]
		Reinforced concrete (RC)	-	[190]
		Fiber Reinforced Concrete (FRC)	Yes	[191]
		Sustainable hybrid fiber reinforced concrete (SHFRC)	Yes	[192]
		Self-compacting concrete (SCC)	-	[153]
	Turnery	Concrete for massive structures	Yes	[137]
	Tires, demolition	Fiber Reinforced Concrete (FRC)	Yes	[193]
Machined steel parts waste	-		-	[71]
Bio-scraps not specified	Agriculture	Fiber Reinforced Concrete (FRC)	Yes	[11]
Carpet	Carpet	Lightweight cementitious composites	Yes	[194]
Cellulose	Algae waste	Fiber Reinforced Concrete (FRC)	Yes	[195]
Coconut coir	Food/steel plant	Concrete	Yes	[196]
Coconut coir	Food	Fiber Reinforced Concrete (FRC)	Yes	[197]
Hair	Human	Fiber Reinforced Concrete (FRC)	Yes	[198]
Hair	Human	Concrete	Yes	[199]
Textile	Textile	Foamed concrete	Yes	[200]
Glass	-	Epoxy polymer concrete with fly ash	-	[201]
Waste glass fiber reinforced polymers (GFRPs)	Waste glass fiber reinforced polymers (GFRPs)	Concrete	-	[202]

## Data Availability

All the data is available within the manuscript.
